# Supplementary material for: Everyday Lives of Middle-Aged Persons with Multimorbidity: A Mixed Methods Systematic Review
Source: Int J Environ Res Public Health. 2021 Dec 21;19(1):6. doi: 10.3390/ijerph19010006 (PMC8751163; doi:10.3390/ijerph19010006)
Supplement: Supplementary file 1 [file ijerph-19-00006-s001.zip › Supplementary files/Figure S1. MuMiA_PRISMA_2020_flow_diagram_new_SRs.pdf]

Figure S1. PRISMA 2020 flow diagram for new systematic reviews which included searches of databases and registers only

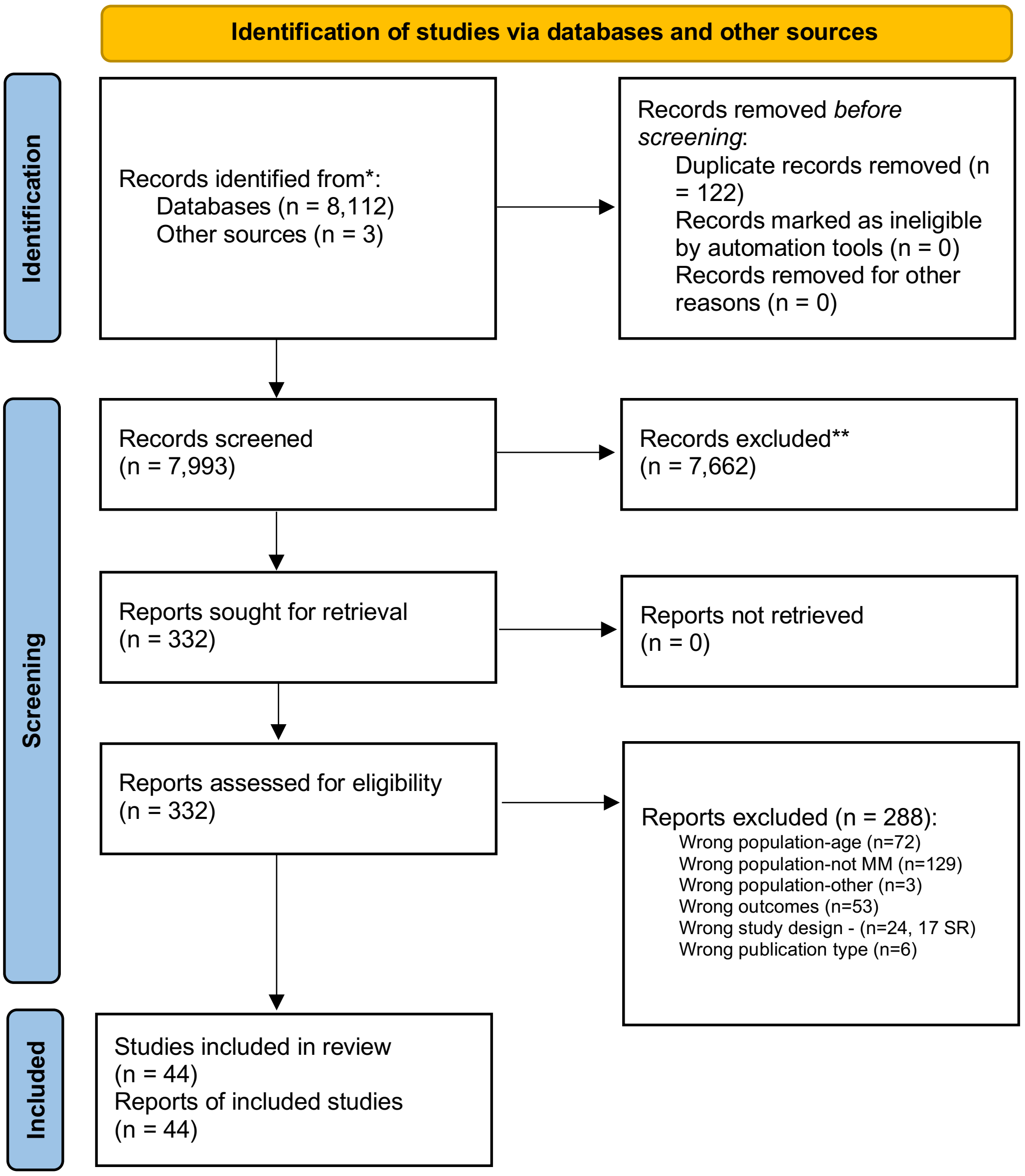

*Consider, if feasible to do so, reporting the number of records identified from each database or register searched (rather than the total number across all databases/registers).

**If automation tools were used, indicate how many records were excluded by a human and how many were excluded by automation tools.

From: Page MJ, McKenzie JE, Bossuyt PM, Boutron I, Hoffmann TC, Mulrow CD, et al. The PRISMA 2020 statement: an updated guideline for reporting systematic reviews. BMJ 2021;372:n71. doi: 10.1136/bmj.n71

For more information, visit: http://www.prisma-statement.org/
