# Supplementary material for: Everyday Lives of Middle-Aged Persons with Multimorbidity: A Mixed Methods Systematic Review
Source: Int J Environ Res Public Health. 2021 Dec 21;19(1):6. doi: 10.3390/ijerph19010006 (PMC8751163; doi:10.3390/ijerph19010006)
Supplement: Supplementary file 1 [file ijerph-19-00006-s001.zip › Supplementary files/Table S3_Exluded studies.pdf]

Table S3. Excluded studies and reasons for exclusion

No.	Authors	Year	Title	Reason for exclusion
1	Abate, T. W.; Gedamu, H.	2020	Psychosocial and clinical factors associated with depression among individuals with diabetes in Bahir Dar City Administrative, Northwest Ethiopia	Wrong outcomes
2	Abraido-Lanza, A. F.; White, K.; Armbrister, A. N.; Link, B. G.	2006	Health status, activity limitations, and disability in work and housework among Latinos and non-Latinos with arthritis: an analysis of national data	Wrong patient population
3	Adeniji, C.; Kenning, C.; Coventry, P. A.; Bower, P.	2015	What are the core predictors of 'hassles' among patients with multimorbidity in primary care? A cross sectional study	Wrong patient population
4	Adepoju, O. E.; Bolin, J. N.; Ohsfeldt, R. L.; Phillips, C. D.; Zhao, H.; Ory, M. G.; Forjuoh, S. N.	2014	Can chronic disease management programs for patients with type 2 diabetes reduce productivity-related indirect costs of the disease? Evidence from a randomized controlled trial	Wrong outcomes
5	Aderka, Idan M.; Hofmann, Stefan G.; Nickerson, Angela; Hermesh, Haggai; Gilboa-Schechtman, Eva; Marom, Sofi	2012	Functional impairment in social anxiety disorder	Wrong patient population
6	Agaliotis, M.; Fransen, M.; Bridgett, L.; Nairn, L.; Votrubec, M.; Jan, S.; Heard, R.; Mackey, M.	2013	Risk factors associated with reduced work productivity among people with chronic knee pain	Wrong outcomes
7	Al-Amer, R.; Ramjan, L.; Glew, P.; Salamonson, Y.	2015	Diagnosis of Type 2 Diabetes: The Experience of Jordanian Patients with Co-existing Depression	Wrong outcomes
8	Alonso, J.; Petukhova, M.; Vilagut, G.; Chatterji, S.; Heeringa, S.; Ustun, T. B.; Alhamzawi, A. O.; Viana, M. C.; Angermeyer, M.; Bromet, E.; Bruffaerts, R.; de Girolamo, G.; Florescu, S.; Gureje, O.; Haro, J. M.; Hinkov, H.; Hu, C. Y.; Karam, E. G.; Kovess, V.; Levinson, D.; Medina-Mora, M. E.; Nakamura, Y.; Ormel, J.; Posada-Villa, J.; Sagar, R.; Scott, K. M.; Tsang, A.; Williams, D. R.; Kessler, R. C.	2011	Days out of role due to common physical and mental conditions: results from the WHO World Mental Health surveys	Wrong patient population
9	Ancker, J. S.; Witteman, H. O.; Hafeez, B.; Provencher, T.; Van de Graaf, M.; Wei, E.	2015	"You Get Reminded You're a Sick Person": Personal Data Tracking and Patients With Multiple Chronic Conditions	Wrong patient population

No.	Authors	Year	Title	Reason for exclusion
10	Andreadou, Elisabeth; Anagnostouli, Maria; Vasdekis, Vassilis; Kararizou, Evangelia; Rentzos, Michael; Kontaxis, Theodoros; Evdokimidis, Ioannis	2011	The impact of comorbidity and other clinical and sociodemographic factors on health-related quality of life in Greek patients with parkinson's disease	Wrong outcomes
11	Anesetti-Rothermel, Andrew; Sambamoorthi, Usha	2011	Physical and mental illness burden: Disability days among working adults	Wrong outcomes
12	Angarita-Osorio, Natalia; Pérez-Aranda, Adrián; Feliu-Soler, Albert; Andrés-Rodríguez, Laura; Borràs, Xavier; Suso-Ribera, Carlos; Slim, Mahmoud; Herrera-Mercadal, Paola; Fernández-Vergel, Rita; Blanco, Mª Elena; Luciano, Juan V.	2020	Patients With Fibromyalgia Reporting Severe Pain but Low Impact of the Syndrome: Clinical and Pain-Related Cognitive Features	Wrong patient population
13	Aras, H. I.; Yazar, M. S.; Atinbas, K.	2013	Quality of life among dually diagnosed and non-substance-using male schizophrenia outpatients	Wrong outcomes
14	Arokiasamy, P.; Uttamacharya, U.; Jain, K.; Biritwum, R. B.; Yawson, A. E.; Wu, F.; Guo, Y.; Maximova, T.; Espinoza, B. M.; Rodriguez, A. S.; Afshar, S.; Pati, S.; Ice, G.; Banerjee, S.; Liebert, M. A.; Snodgrass, J. J.; Naidoo, N.; Chatterji, S.; Kowal, P.	2015	The impact of multimorbidity on adult physical and mental health in low- and middle-income countries: what does the study on global ageing and adult health (SAGE) reveal?	Wrong patient population
15	Arreskov, A. B.; Graungaard, A. H.; Kristensen, M. T.; Sondergaard, J.; Davidsen, A. S.	2018	Life goes on... Patient perspectives on having a cancer diagnosis and other comorbid diseases: A qualitative study	Wrong patient population
16	Badcock, Johanna C.; Mackinnon, Andrew; Waterreus, Anna; Watts, Gerald F.; Castle, David; McGrath, John J.; Morgan, Vera A.	2019	Loneliness in psychotic illness and its association with cardiometabolic disorders	Wrong patient population
17	Bae, S. C.; Hashimoto, H.; Karlson, E. W.; Liang, M. H.; Daltroy, L. H.	2001	Variable effects of social support by race, economic status, and disease activity in systemic lupus erythematosus	Wrong patient population
18	Bailey, Ryan R.; Birkenmeier, Rebecca L.; Lang, Catherine E.	2015	Real-world affected upper limb activity in chronic stroke: An examination of potential modifying factors	Wrong patient population

No.	Authors	Year	Title	Reason for exclusion
19	Bair, M. J.; Matthias, M. S.; Nyland, K. A.; Huffman, M. A.; Stubbs, D. L.; Kroenke, K.; Damush, T. M.	2009	Barriers and facilitators to chronic pain self-management: a qualitative study of primary care patients with comorbid musculoskeletal pain and depression	Wrong patient population
20	Baker, Shula; McBeth, John; Chew-Graham, Carolyn A.; Wilkie, Ross	2017	Musculoskeletal pain and co-morbid insomnia in adults; a population study of the prevalence and impact on restricted social participation	Wrong patient population
21	Bardach, S. H.; Tarasenko, Y. N.; Schoenberg, N. E.	2011	The role of social support in multiple morbidity: self-management among rural residents	Wrong patient population
22	Barlow, J. H.; Wright, C. C.; Williams, B.; Keat, A.	2001	Work disability among people with ankylosing spondylitis	Wrong patient population
23	Bartlett, S. J.; Lambert, S. D.; McCusker, J.; Yaffe, M.; de Raad, M.; Belzile, E.; Ciampi, A.; Di Carlo, M.; Lyddiatt, A.	2020	Self-management across chronic diseases: Targeting education and support needs	Wrong outcomes
24	Bassett, S. S.; Chase, G. A.; Folstein, M. F.; Regier, D. A.	1998	Disability and psychiatric disorders in an urban community: measurement, prevalence and outcomes	Wrong outcomes
25	Baune, B. T.; Adrian, I.; Jacobi, F.	2007	Medical disorders affect health outcome and general functioning depending on comorbid major depression in the general population	Wrong patient population
26	Bayliss, E. A.; Steiner, J. F.; Fernald, D. H.; Crane, L. A.; Main, D. S.	2003	Descriptions of barriers to self-care by persons with comorbid chronic diseases	Wrong patient population
27	Bernard, Renaldo; Sabariego, Carla; Cieza, Alarcos	2019	Difficulties encountered by people with depression and anxiety on the web: Qualitative study and web-based expert survey	Wrong outcomes
28	Bertera, E. M.	2005	Mental health in U.S. adults: the role of positive social support and social negativity in personal relationships	Wrong patient population
29	Bijl, R. V.; Ravelli, A.	2000	Current and residual functional disability associated with psychopathology: findings from the Netherlands Mental Health Survey and Incidence Study (NEMESIS)	Wrong outcomes
30	Boeckxstaens, P.; Deregt, M.; Vandesype, P.; Willems, S.; Brusselle, G.; De Sutter, A.	2012	Chronic obstructive pulmonary disease and comorbidities through the eyes of the patient	Wrong patient population

No.	Authors	Year	Title	Reason for exclusion
31	Boeckxstaens, P.; Willems, S.; Lanssens, M.; Decuypere, C.; Brusselle, G.; Kuhlein, T.; De Maeseneer, J.; De Sutter, A.	2016	A qualitative interpretation of challenges associated with helping patients with multiple chronic diseases identify their goals	Wrong patient population
32	Boehm, A.; Eisenberg, E.; Lampel, S.	2011	The contribution of social capital and coping strategies to functioning and quality of life of patients with fibromyalgia	Wrong patient population
33	Boehmer, K. R.; Gionfriddo, M. R.; Rodriguez-Gutierrez, R.; Dabrh, A. M.; Leppin, A. L.; Hargraves, I.; May, C. R.; Shippee, N. D.; Castaneda-Guarderas, A.; Palacios, C. Z.; Bora, P.; Erwin, P.; Montori, V. M.	2016	Patient capacity and constraints in the experience of chronic disease: a qualitative systematic review and thematic synthesis	Wrong study design
34	Bolge, S. C.; Joish, V. N.; Balkrishnan, R.; Kannan, H.; Drake, C. L.	2010	Burden of chronic sleep maintenance insomnia characterized by nighttime awakenings among anxiety and depression sufferers: results of a national survey	Wrong patient population
35	Bosire, E. N.; Mendenhall, E.; Weaver, L. J.	2020	Comorbid Suffering: Breast Cancer Survivors in South Africa	Wrong study design
36	Bounthavong, M.; Law, A. V.	2008	Identifying health-related quality of life (HRQL) domains for multiple chronic conditions (diabetes, hypertension and dyslipidemia): patient and provider perspectives	Wrong patient population
37	Bower, P.; Hann, M.; Rick, J.; Rowe, K.; Burt, J.; Roland, M.; Protheroe, J.; Richardson, G.; Reeves, D.	2013	Multimorbidity and delivery of care for long-term conditions in the English National Health Service: baseline data from a cohort study	Wrong patient population
38	Braden, J. B.; Zhang, L.; Zimmerman, F. J.; Sullivan, M. D.	2008	Employment outcomes of persons with a mental disorder and comorbid chronic pain	Wrong patient population
39	Breek, J. C.; Hamming, J. F.; De Vries, J.; Henegouwen, D. P. V.; van Heck, G. L.	2002	The impact of walking impairment, cardiovascular risk factors, and comorbidity on quality of life in patients with intermittent claudication	Wrong patient population
40	Breslin, F. C.; Gnam, W.; Franche, R. L.; Mustard, C.; Lin, E.	2006	Depression and activity limitations: examining gender differences in the general population	Wrong patient population
41	Bronisch, Thomas; Hecht, Heidemarie	1990	Major depression with and without a coexisting anxiety disorder: social dysfunction, social integration, and personality features	Wrong outcomes
42	Browne, J. L.; Scibilia, R.; Speight, J.	2013	The needs, concerns, and characteristics of younger Australian adults with Type 2 diabetes	Wrong outcomes

No.	Authors	Year	Title	Reason for exclusion
43	Bruffaerts, R.; Vilagut, G.; Demyttenaere, K.; Alonso, J.; AlHamzawi, A.; Andrade, L. H.; Benjet, C.; Bromet, E.; Bunting, B.; de Girolamo, G.; Florescu, S.; Gureje, O.; Haro, J. M.; He, Y. L.; Hinkov, H.; Hu, C. Y.; Karam, E. G.; Lepine, J. P.; Levinson, D.; Matschinger, H.; Nakane, Y.; Ormel, J.; Posada-Villa, J.; Scott, K. M.; Varghese, M.; Williams, D. R.; Xavier, M.; Kessler, R. C.	2012	Role of common mental and physical disorders in partial disability around the world	Wrong patient population
44	Buck, H. G.; Meghani, S.; Bettger, J. P.; Byun, E.; Fachko, M. J.; O'Connor, M.; Tocchi, C.; Naylor, M.	2012	The use of comorbidities among adults experiencing care transitions: a systematic review and evolutionary analysis of empirical literature	Wrong study design
45	Bugajski, A.; Frazier, S. K.; Moser, D. K.; Lennie, T. A.; Chung, M.	2019	Psychometric testing of the Multidimensional Scale of Perceived Social Support in patients with comorbid COPD and heart failure	Wrong patient population
46	Cabrera-Leon, A.; Cantero-Braojos, M. A.; Garcia-Fernandez, L.; Guerra de Hoyos, J. A.	2018	Living with disabling chronic pain: results from a face-to-face cross-sectional population-based study	Wrong outcomes
47	Cairney, J.; Corna, L. M.; Veldhuizen, S.; Herrmann, N.; Streiner, D. L.	2008	Comorbid depression and anxiety in later life: patterns of association, subjective well-being, and impairment	Wrong patient population
48	Carlisle, A. C.; John, A. M.; Fife-Schaw, C.; Lloyd, M.	2005	The self-regulatory model in women with rheumatoid arthritis: relationships between illness representations, coping strategies, and illness outcome	Wrong patient population
49	Catalina-Romero, C.; Martinez-Munoz, P.; Quevedo-Aguado, L.; Ruiz-Moraga, M.; Fernandez-Labandera, C.; Calvo-Bonacho, E.	2013	Predictors of the duration of non-work-related sick leave due to anxiety disorders	Wrong patient population
50	Cavers, D.; Habets, L.; Cunningham-Burley, S.; Watson, E.; Banks, E.; Campbell, C.	2019	Living with and beyond cancer with comorbid illness: a qualitative systematic review and evidence synthesis	Wrong study design
51	Cella, M.; Sharpe, M.; Chalder, T.; Cella, Matteo; Sharpe, Michael; Chalder, Trudie	2011	Measuring disability in patients with chronic fatigue syndrome: reliability and validity of the Work and Social Adjustment Scale	Wrong outcomes
52	Cerrato, A.; Avitable, M.; Hayman, L. L.	2008	The relationship between the sick role and functional ability: one center's experience	Wrong patient population

No.	Authors	Year	Title	Reason for exclusion
53	Chakraborty, S.; Subramanya, A. H.	2013	Socio-demographic and clinical predictors of absenteeism - A cross-sectional study of urban industrial employees	Wrong patient population
54	Chen, H. M.; Clark, A. P.; Tsai, L. M.; Chao, Y. F.	2009	Self-reported sleep disturbance of patients with heart failure in Taiwan	Wrong patient population
55	Cheng, C.; Inder, K.; Chan, S. W.	2020	Coping with multiple chronic conditions: An integrative review	Wrong study design
56	Cheng, C.; Inder, K.; Chan, S. W.	2019	Patients' experiences of coping with multiple chronic conditions: A meta-ethnography of qualitative work	Wrong study design
57	Cheng, L.; Cumber, S.; Dumas, C.; Winter, R.; Nguyen, K. M.; Nieman, L. Z.	2003	Health related quality of life in pregeriatric patients with chronic diseases at urban, public supported clinics	Wrong patient population
58	Cho, E.; Chen, T. Y.	2018	The Effects of Work-Family Experiences on Health Among Older Workers	Wrong patient population
59	Churcher, L.; Chan, C. H.; Badley, E. M.	2013	Chronic back problems and labor force participation in a national population survey: impact of comorbid arthritis	Wrong patient population
60	Cook, J. A.; Razzano, L. A.; Burke-Miller, J. K.; Blyler, C. R.; Leff, H. S.; Mueser, K. T.; Gold, P. B.; Goldberg, R. W.; Shafer, M. S.; Onken, S. J.; McFarlane, W. R.; Donegan, K.; Carey, M. A.; Kaufmann, C.; Grey, D. D.	2007	Effects of co-occurring disorders on employment outcomes in a multisite randomized study of supported employment for people with severe mental illness	Wrong patient population
61	Coventry, P. A.; Fisher, L.; Kenning, C.; Bee, P.; Bower, P.	2014	Capacity, responsibility, and motivation: a critical qualitative evaluation of patient and practitioner views about barriers to self-management in people with multimorbidity	Wrong patient population
62	Coventry, P. A.; Small, N.; Panagioti, M.; Adeyemi, I.; Bee, P.	2015	Living with complexity; marshalling resources: a systematic review and qualitative meta-synthesis of lived experience of mental and physical multimorbidity	Wrong study design
63	Coyne, K. S.; Wein, A.; Nicholson, S.; Kvasz, M.; Chen, C. I.; Milsom, I.	2013	Comorbidities and personal burden of urgency urinary incontinence: a systematic review	Wrong study design
64	Crisp, R.	2007	Depression and occupational disability in five diagnostic groups: a review of recent research	Wrong study design

No.	Authors	Year	Title	Reason for exclusion
65	Cunningham, P. J.; Green, T. L.; Braun, R. T.	2018	Income Disparities in the Prevalence, Severity, and Costs of Co-occurring Chronic and Behavioral Health Conditions	Wrong outcomes
66	Dale, C. M.; Angus, J. E.; Seto Nielsen, L.; Kramer-Kile, M.; Pritlove, C.; Lapum, J.; Price, J.; Marzolini, S.; Abramson, B.; Oh, P.; Clark, A.	2015	"I'm No Superman": Understanding Diabetic Men, Masculinity, and Cardiac Rehabilitation	Wrong patient population
67	Das-Munshi, J.; Stewart, R.; Ismail, K.; Bebbington, P. E.; Jenkins, R.; Prince, M. J.	2007	Diabetes, common mental disorders, and disability: findings from the UK National Psychiatric Morbidity Survey	Wrong patient population
68	de Munter, L.; Geraerds, Ajlm; de Jongh, M. A. C.; van der Vlegel, M.; Steyerberg, E. W.; Haagsma, J. A.; Polinder, S.	2020	Prognostic factors for medical and productivity costs, and return to work after trauma	Wrong outcomes
69	Dennis, S. M.; Harris, M.; Lloyd, J.; Powell Davies, G.; Faruqi, N.; Zwar, N.	2013	Do people with existing chronic conditions benefit from telephone coaching? A rapid review	Wrong study design
70	Dewa, Carolyn S.; Lin, Elizabeth; Kooehoorn, Mieke; Goldner, Elliot	2007	Association of chronic work stress, psychiatric disorders, and chronic physical conditions with disability among workers	Wrong patient population
71	Dismuke, C. E.; Egede, L. E.	2010	Association between major depression, depressive symptoms and personal income in US adults with diabetes	Wrong outcomes
72	Druss, B. G.; Marcus, S. C.; Rosenheck, R. A.; Olfson, M.; Tanielian, T.; Pincus, H. A.	2000	Understanding disability in mental and general medical conditions	Wrong patient population
73	Duffield, S. J.; Ellis, B. M.; Goodson, N.; Walker-Bone, K.; Conaghan, P. G.; Margham, T.; Loftis, T.	2017	The contribution of musculoskeletal disorders in multimorbidity: Implications for practice and policy	Wrong study design
74	Eapen, Valsamma; Cavanna, Andrea E.; Robertson, Mary M.	2016	Comorbidities, social impact, and quality of life in Tourette syndrome	Wrong puplucation type (e.g. conference proceeding)
75	Egede, L. E.; Grubaugh, A. L.; Ellis, C.	2010	The effect of major depression on preventive care and quality of life among adults with diabetes	Wrong outcomes

No.	Authors	Year	Title	Reason for exclusion
76	Emptage, N. P.; Sturm, R.; Robinson, R. L.	2005	Depression and comorbid pain as predictors of disability, employment, insurance status, and health care costs	Wrong patient population
77	Ennis, Edel; O'Neill, S.; Murphy, S.; Bunting, B.	2016	Days out of role due to common physical and mental conditions: Results from the Northern Ireland study of health and stress	Wrong patient population
78	Ephraim, P. L.; MacKenzie, E. J.; Wegener, S. T.; Dillingham, T. R.; Pezzin, L. E.	2006	Environmental barriers experienced by amputees: the Craig Hospital Inventory of Environmental Factors-Short Form	Wrong patient population
79	Eppler, Natalie; Kuplewatzky, Nina	2010	Lebensqualität trotz Multimorbidität? Wie ältere DrogenkonsumentInnen mit Erkrankungen umgehen	Wrong puplucation type (e.g. conference proceeding)
80	Eren, I.; Erdi, O.; Sahin, M.	2008	The effect of depression on quality of life of patients with type II diabetes mellitus	Wrong outcomes
81	Ervasti, J.; Kivimäki, M.; Dray-Spira, R.; Head, J.; Goldberg, M.; Pentti, J.; Jokela, M.; Vahtera, J.; Zins, M.; Virtanen, M.	2016	Psychosocial factors associated with work disability in men and women with diabetes: a pooled analysis of three occupational cohort studies	Wrong patient population
82	Ervasti, Jenni; Joensuu, Matti; Pentti, Jaana; Oksanen, Tuula; Ahola, Kirsi; Vahtera, Jussi; Kivimäki, Mika; Virtanen, Marianna	2017	Prognostic factors for return to work after depression-related work disability: A systematic review and meta-analysis	Wrong study design
83	Ervasti, Jenni; Kivimäki, Mika; Dray-Spira, Rosemary; Head, Jenny; Goldberg, Marcel; Pentti, Jaana; Jokela, Markus; Vahtera, Jussi; Zins, Marie; Virtanen, Marianna	2016	Socioeconomic gradient in work disability in diabetes: evidence from three occupational cohorts	Wrong patient population
84	Esposito, Eleonora; Wang, Jian Li; Williams, Jeanne V. A.; Patten, Scott B.	2007	Mood and anxiety disorders, the association with presenteeism in employed members of a general population sample	Wrong patient population
85	Ettinger, A.; Reed, M.; Cramer, J.; Epilepsy Impact Project, Group	2004	Depression and comorbidity in community-based patients with epilepsy or asthma	Wrong patient population
86	Everhov, A. H.; Khalili, H.; Askling, J.; Myrelid, P.; Ludvigsson, J. F.; Halfvarson, J.;	2019	Work Loss Before and After Diagnosis of Crohn's Disease	Wrong patient population

No.	Authors	Year	Title	Reason for exclusion
	Nordenvall, C.; Neovius, M.; Soderling, J.; Olen, O.			
87	Flynn, S.; Hulbert-Williams, N. J.; Hulbert-Williams, L.; Bramwell, R.	2016	"You don't know what's wrong with you": an exploration of cancer-related experiences in people with an intellectual disability	Wrong patient population
88	Fortenbaugh, Francesca C.; Fonda, Jennifer R.; Fortier, Catherine B.; Amick, Melissa M.; Milberg, William P.; McGlinchey, Regina E.	2020	The Impact of Common Psychiatric and Behavioral Comorbidities on Functional Disability Across Time and Individuals in Post-9/11 Veterans	Wrong patient population
89	Fortin, M.; Dubois, M. F.; Hudon, C.; Soubhi, H.; Almirall, J.	2007	Multimorbidity and quality of life: a closer look	Wrong outcomes
90	Fouad, A. M.; Waheed, A.; Gamal, A.; Amer, S. A.; Abdellah, R. F.; Shebl, F. M.	2017	Effect of Chronic Diseases on Work Productivity A Propensity Score Analysis	Wrong patient population
91	Francis, Helen; Carryer, Jenny; Wilkinson, Jill	2019	Patient expertise: Contested territory in the realm of long-term condition care	Wrong patient population
92	Frank, A. O.; De Souza, L. H.; McAuley, J. H.; Sharma, V.; Main, C. J.	2000	A cross-sectional survey of the clinical and psychological features of low back pain and consequent work handicap: use of the Quebec Task Force classification	Wrong patient population
93	Fry, M.; McLachlan, S.; Purdy, S.; Sanders, T.; Kadam, U. T.; Chew-Graham, C. A.	2016	The implications of living with heart failure; the impact on everyday life, family support, co-morbidities and access to healthcare: a secondary qualitative analysis	Wrong patient population
94	Fu, A. Z.; Qiu, Y.; Radican, L.; Luo, N.	2011	Marginal differences in health-related quality of life of diabetic patients with and without macrovascular comorbid conditions in the United States	Wrong patient population
95	Fu, A. Z.; Qiu, Y.; Radican, L.; Wells, B. J.	2009	Health care and productivity costs associated with diabetic patients with macrovascular comorbid conditions	Wrong patient population
96	Gamma, Alex; Angst, Jules	2001	Concurrent psychiatric comorbidity and multimorbidity in a community study: gender differences and quality of life	Wrong outcomes
97	Gask, L.; Macdonald, W.; Bower, P.	2011	What is the relationship between diabetes and depression? a qualitative meta-synthesis of patient experience of co-morbidity	Wrong study design
98	Ge, L.; Yap, C. W.; Heng, B. H.	2018	Sex differences in associations between multimorbidity and physical function domains among community-dwelling adults in Singapore	Wrong patient population

No.	Authors	Year	Title	Reason for exclusion
99	Ginzburg, K.; Ein-Dor, T.; Solomon, Z.	2010	Comorbidity of posttraumatic stress disorder, anxiety and depression: a 20-year longitudinal study of war veterans	Wrong outcomes
100	Glozier, N.; Hackett, M. L.; Parag, V.; Anderson, C. S.; Auckland Regional Community Stroke Study, Group	2008	The influence of psychiatric morbidity on return to paid work after stroke in younger adults: the Auckland Regional Community Stroke (ARCOS) Study, 2002 to 2003	Wrong patient population
101	Gonah, L.; Moodley, I.; Hlongwana, K.	2020	Effects of HIV and non-communicable disease comorbidity on healthcare costs and health experiences in people living with HIV in Zimbabwe	Wrong patient population
102	Goodwin, R. D.; Pagura, J.; Cox, B.; Sareen, J.	2010	Asthma and mental disorders in Canada: impact on functional impairment and mental health service use	Wrong patient population
103	Hajak, G.; Petukhova, M.; Lakoma, M. D.; Coulouvrat, C.; Roth, T.; Sampson, N. A.; Shahly, V.; Shillington, A. C.; Stephenson, J. J.; Walsh, J. K.; Kessler, R. C.	2011	Days-out-of-role associated with insomnia and comorbid conditions in the America Insomnia Survey	Wrong outcomes
104	Hansen, S. M.; Hetland, M. L.; Pedersen, J.; Ostergaard, M.; Rubak, T. S.; Bjorner, J. B.	2017	Work ability in rheumatoid arthritis patients: a register study on the prospective risk of exclusion and probability of returning to work	Wrong patient population
105	Hartge, Joseph; Toledo, Patricia	2018	Attention deficit hyperactivity disorder (ADHD) and its comorbid mental disorders: An evaluation of their labor market outcomes	Wrong patient population
106	Hassett, M. J.; O'Malley, A. J.; Keating, N. L.	2009	Factors Influencing Changes in Employment Among Women With Newly Diagnosed Breast Cancer	Wrong patient population
107	Hauser, W.; Wolfe, F.; Henningsen, P.; Schmutzer, G.; Brahler, E.; Hinz, A.	2014	Untying chronic pain: prevalence and societal burden of chronic pain stages in the general population - a cross-sectional survey	Wrong patient population
108	Hecht, Heidemarie; von Zerssen, Detlev; Wittchen, Hans-Ulrich	1990	Anxiety and depression in a community sample: The influence of comorbidity on social functioning (PSYNDEXshort)	Wrong patient population
109	Henry, C.; Gustafson, C.; Paul, S.; Plantinga, L.; Turberville-Trujillo, L.; Ojeniyi, O. O.; Song, M. K.	2019	SOCIAL NETWORKS OF SELF-MANAGEMENT AND CARE COORDINATION AMONG PATIENTS ON DIALYSIS	Wrong outcomes
110	Heo, S.; Lennie, T. A.; Okoli, C.; Moser, D. K.	2009	Quality of life in patients with heart failure: ask the patients	Wrong outcomes
111	Heo, Seongkum; Moser, Debra K.; Lennie, Terry A.; Riegel, Barbara; Chung, Misook L.	2008	Gender differences in and factors related to self-care behaviors: A cross-sectional, correlational study of patients with heart failure	Wrong patient population

No.	Authors	Year	Title	Reason for exclusion
112	Herzig, Lilli; Zeller, Andreas; Pasquier, Jérôme; Streit, Sven; Neuner-Jehle, Stefan; Excoffier, Sophie; Haller, Dagmar M.	2019	Factors associated with patients' and GPs' assessment of the burden of treatment in multimorbid patients: a cross-sectional study in primary care	Wrong patient population
113	Hilari, Katerina; Wiggins, Richard D.; Roy, Penny; Byng, Sally; Smith, Sara C.	2003	Predictors of health-related quality of life (HRQL) in people with chronic aphasia	Wrong patient population
114	Ho, CSh; Feng, L.; Fam, J.; Mahendran, R.; Kua, E. H.; Ng, T. P.	2014	Coexisting medical comorbidity and depression: multiplicative effects on health outcomes in older adults	Wrong patient population
115	Ho, H. Y.; Chen, M. H.; Lou, M. F.	2018	Exploring the experiences of older Chinese adults with comorbidities including diabetes: surmounting these challenges in order to live a normal life	Wrong patient population
116	Ho, J. W.; Kuluski, K.; Im, J.	2017	"It's a fight to get anything you need" - Accessing care in the community from the perspectives of people with multimorbidity	Wrong patient population
117	Holden, L.; Scuffham, P. A.; Hilton, M. F.; Ware, R. S.; Vecchio, N.; Whiteford, H. A.	2011	Health-related productivity losses increase when the health condition is co-morbid with psychological distress: findings from a large cross-sectional sample of working Australians	Wrong patient population
118	Horn, A. B.; Boettcher, V. S.; Holzer, B. M.; Siebenhuener, K.; Maercker, A.; Battegay, E.; Zimmerli, L.	2019	Couples Adjusting to Multimorbidity: A Dyadic Study on Disclosure and Adjustment Disorder Symptoms	Wrong patient population
119	Hreha, Kimberly P.; Smith, Amanda E.; Wong, Jennifer L.; Mroz, Tracy M.; Fogelberg, Donald J.; Molton, Ivan	2019	Impact of secondary health conditions on social role participation for a long-term physical disability cohort	Wrong patient population
120	Huang, Hsiang; Russo, Joan; Von Korff, Michael; Ciechanowski, Paul; Lin, Elizabeth; Ludman, Evette; Katon, Wayne	2012	The effect of changes in depressive symptoms on disability status in patients with diabetes	Wrong patient population
121	Iancu, S. C.; Batelaan, N. M.; Zweekhorst, M. B.; Bunders, J. F.; Veltman, D. J.; Penninx, B. W.; van Balkom, A. J.	2014	Trajectories of functioning after remission from anxiety disorders: 2-year course and outcome predictors	Wrong patient population
122	Irfan Khan, A.; Gill, A.; Cott, C.; Hans, P. K.; Steele Gray, C.	2018	mHealth Tools for the Self-Management of Patients With Multimorbidity in Primary Care Settings: Pilot Study to Explore User Experience	Wrong outcomes
123	Ivanova, J. I.; Birnbaum, H. G.; Samuels, S.; Davis, M.; Phillips, A. L.; Meletiche, D.;	2009	The cost of disability and medically related absenteeism among employees with multiple sclerosis in the US	Wrong patient population

No.	Authors	Year	Title	Reason for exclusion
	Ivanova, Jasmina I.; Birnbaum, Howard G.; Samuels, Seth; Davis, Matthew; Phillips, Amy L.; Meletiche, Dennis			
124	Jayathilaka, R.; Joachim, S.; Mallikarachchi, V.; Perera, N.; Ranawaka, D.	2020	Chronic diseases: An added burden to income and expenses of chronically-ill people in Sri Lanka	Wrong outcomes
125	Johnson-Lawrence, V.; Zajacova, A.; Sneed, R.	2017	Education, race/ethnicity, and multimorbidity among adults aged 30-64 in the National Health Interview Survey	Wrong outcomes
126	Judd, L. L.; Kessler, R. C.; Paulus, M. P.; Zeller, P. V.; Wittchen, H. U.; Kunovac, J. L.	1998	Comorbidity as a fundamental feature of generalized anxiety disorders: results from the National Comorbidity Study (NCS)	Wrong study design
127	Kadam, U. T.; Croft, P. R.; North Staffordshire, G. P. Consortium Group	2007	Clinical multimorbidity and physical function in older adults: a record and health status linkage study in general practice	Wrong patient population
128	Kafkia, Theodora; Vehvilainen-Julkunen, Katri; Sapountzi-Krepia, Despina	2017	Renal Patients' Quality of Life as it is Affected by Pain	Wrong study design
129	Kehbila, J.; Ekabe, C. J.; Aminde, L. N.; Noubiap, J. J.; Fon, P. N.; Monekosso, G. L.	2016	Prevalence and correlates of depressive symptoms in adult patients with pulmonary tuberculosis in the Southwest Region of Cameroon	Wrong outcomes
130	Kennedy, M.; Papneja, A.; Thavaneswaran, A.; Chandran, V.; Gladman, D. D.	2014	Prevalence and predictors of reduced work productivity in patients with psoriatic arthritis	Wrong patient population
131	Kenning, C.; Coventry, P. A.; Gibbons, C.; Bee, P.; Fisher, L.; Bower, P.	2015	Does patient experience of multimorbidity predict self-management and health outcomes in a prospective study in primary care?	Wrong patient population
132	Kessler, R. C.; Greenberg, P. E.; Mickelson, K. D.; Meneades, L. M.; Wang, P. S.	2001	The effects of chronic medical conditions on work loss and work cutback	Wrong patient population
133	Kinoshita, Y.; Dibonaventura, M.; Rossi, B.; Iwamoto, K.; Wang, E. C.; Briere, J. B.	2013	Burden of comorbidities among Japanese patients with atrial fibrillation: a case study of dyspepsia	Wrong patient population
134	Kivimaki, M.; Vahtera, J.; Pentti, J.; Virtanen, M.; Elovainio, M.; Hemingway, H.	2007	Increased sickness absence in diabetic employees: what is the role of co-morbid conditions?	Wrong patient population

No.	Authors	Year	Title	Reason for exclusion
135	Kleinman, N. L.; Brook, R. A.; Patel, P. A.; Melkonian, A. K.; Brizee, T. J.; Smeeding, J. E.; Joseph-Ridge, N.	2007	The impact of gout on work absence and productivity	Wrong patient population
136	Korolainen, M. A.; Kurki, S.; Lassenius, M. I.; Toppila, I.; Costa-Scharplatz, M.; Purmonen, T.; Nissila, M.	2019	Burden of migraine in Finland: health care resource use, sick-leaves and comorbidities in occupational health care	Wrong patient population
137	Koster, Annemarie; Bosma, Hans; Kempen, Gertrudis I. J. M.; van Lenthe, Frank J.; van Eijk, Jacques Th M.; Mackenbach, Johan P.	2004	Socioeconomic inequalities in mobility decline in chronic disease groups (asthma/COPD, heart disease, diabetes mellitus, low back pain): Only a minor role for disease severity and comorbidity	Wrong patient population
138	Kotiranta, Ulla; Forssell, Heli; Kauppila, Timo	2019	Painful temporomandibular disorders (TMD) and comorbidities in primary care: associations with pain-related disability	Wrong patient population
139	Kourlaba, G.; Hillas, G.; Vassilakopoulos, T.; Maniadakis, N.	2016	The disease burden of chronic obstructive pulmonary disease in Greece	Wrong patient population
140	Lackner, Jeffrey M.; Gudleski, Gregory D.; Firth, Rebecca; Keefer, Laurie; Brenner, Darren M.; Guy, Katie; Simonetti, Camille; Radziwon, Christopher; Quinton, Sarah; Krasner, Susan S.; Katz, Leonard; Garbarino, Guido; Iacobucci, Gary D.; Sitrin, Michael D.	2013	Negative aspects of close relationships are more strongly associated than supportive personal relationships with illness burden of irritable bowel syndrome	Wrong patient population
141	Lackner, Johanna B.; Joseph, Jill G.; Ostrow, David G.; Eshleman, Suzann	1993	The effects of social support on Hopkins Symptom Checklist-assessed depression and distress in a cohort of human immunodeficiency virus-positive and -negative gay men: A longitudinal study at six time points	Wrong patient population
142	Lai, F. T. T.; Ma, T. W.; Hou, W. K.	2020	How does chronic multimorbidity affect daily routines? An experience sampling study of community-dwelling adults in Hong Kong	Wrong patient population
143	Larson, Mary Jo; Miller, Lisa; Becker, Marion; Richardson, Erin; Kammerer, Nina; Thom, Jennifer; Gampel, Joanne; Savage, Andrea	2005	Physical Health Burdens of Women With Trauma Histories and Co-occurring Substance Abuse and Mental Disorders	Wrong patient population
144	Lavigne, J. E.; Phelps, C. E.; Mushlin, A.; Lednar, W. M.	2003	Reductions in individual work productivity associated with type 2 diabetes mellitus	Wrong patient population

No.	Authors	Year	Title	Reason for exclusion
145	Lee, S.; Mendelsohn, A.; Sarnes, E.	2010	The burden of psoriatic arthritis: a literature review from a global health systems perspective	Wrong study design
146	Levy, C.; Balogh, S.; Perkins, E.	2016	Realizing the Potential of Rehabilitative Care for People with Complex Health Conditions: The Time Is Now	Wrong publication type (e.g. conference proceeding)
147	Liddy, C.; Blazkho, V.; Mill, K.	2014	Challenges of self-management when living with multiple chronic conditions: systematic review of the qualitative literature	Wrong study design
148	Lillie, E.; Alvarado, B. E.; Stuart, H.	2013	Unemployment among Canadians with physical and a co-morbid mental disability: an examination of the 2006 Participation and Activity Limitation Survey (PALS)	Wrong patient population
149	Lindbohm, M. L.; Taskila, T.; Kuosma, E.; Hietanen, P.; Carlsen, K.; Gudbergsson, S.; Gunnarsdottir, H.	2012	Work ability of survivors of breast, prostate, and testicular cancer in Nordic countries: a NOCWO study	Wrong patient population
150	Liu, J. C.; Chang, L. Y.; Wu, S. Y.; Tsai, P. S.	2015	Resilience mediates the relationship between depression and psychological health status in patients with heart failure: a cross-sectional study	Wrong patient population
151	Liu, X.; Liu, C.; Tian, X.; Zou, G.; Li, G.; Kong, L.; Li, P.	2016	Associations of Perceived Stress, Resilience and Social Support with Sleep Disturbance Among Community-dwelling Adults	Wrong patient population
152	Livingstone, Wendy; Van De Mortel, Thea F.; Taylor, Beverly	2011	A path of perpetual resilience: Exploring the experience of a diabetes-related amputation through grounded theory	Wrong patient population
153	Löffler, Christin; Altiner, Attila; Streich, Waldemar; Stolzenbach, Carl-Otto; Fuchs, Angela; Drewelow, Eva; Hornung, Anne; Feldmeier, Gregor; van den Bussche, Hendrik; Kaduszkiewicz, Hanna	2015	Multimorbidität aus Hausarzt-und Patientensicht: Qualitative Studie. = Approaches of general practitioners and patients to multimorbidity: Qualitative study	Wrong study design
154	Löwe, Bernd; Kroenke, Kurt; Spitzer, Robert L.; Williams, Janet B. W.; Mussell, Monika;	2011	Trauma exposure and posttraumatic stress disorder in primary care patients: Cross-sectional criterion standard study	Wrong patient population

No.	Authors	Year	Title	Reason for exclusion
	Wingenfeld, Katja; Sauer, Nina; Spitzer, Carsten			
155	Luijks, H. D.; Lagro-Janssen, A. L. M.; van Weel, C.	2016	Multimorbidity and the primary healthcare perspective	Wrong publication type (e.g. conference proceeding)
156	Macy, A. S.; Theo, J. N.; Kaufmann, S. C. V.; Ghazzaoui, R. B.; Pawlowski, P. A.; Fakhry, H. I.; Cassmassi, B. J.; IsHak, W. W.	2013	Quality of life in obsessive compulsive disorder	Wrong patient population
157	Maduka, S.; Tudorascu, D. L.; McTigue, K. M.; Bryce, C. L.; Huber, K. A.; Simkin-Silverman, L. R.; Comer, D.; Hess, R.; Fischer, G.; Conroy, M. B.	2016	Perceptions of social support in persons with medical comorbidities and associations with intentional weight loss: results from the maintain-PC study	Wrong publication type (e.g. conference proceeding)
158	Makovski, T. T.; Le Coroller, G.; Putrik, P.; Choi, Y. H.; Zeegers, M. P.; Stranges, S.; Ruiz Castell, M.; Huiart, L.; van den Akker, M.	2020	Role of clinical, functional and social factors in the association between multimorbidity and quality of life: Findings from the Survey of Health, Ageing and Retirement in Europe (SHARE)	Wrong patient population
159	Manderson, Lenore; Kokanovic, Renata	2009	'Worried all the time': Distress and the circumstances of everyday life among immigrant Australians with type 2 diabetes	Wrong patient population
160	Mason, B.; Nanton, V.; Epiphaniou, E.; Murray, S. A.; Donaldson, A.; Shipman, C.; Daveson, B. A.; Harding, R.; Higginson, I. J.; Munday, D.; Barclay, S.; Dale, J.; Kendall, M.; Worth, A.; Boyd, K.	2016	'My body's falling apart.' Understanding the experiences of patients with advanced multimorbidity to improve care: serial interviews with patients and carers	Wrong patient population
161	Matza, Louis S.; Buchanan, Robert; Purdon, Scot; Brewster-Jordan, Jessica; Zhao, Yang; Revicki, Dennis A.	2006	Measuring Changes in Functional Status Among Patients With Schizophrenia: The Link With Cognitive Impairment	Wrong study design
162	McDaid, O.; Hanly, M. J.; Richardson, K.; Kee, F.; Kenny, R. A.; Savva, G. M.	2013	The effect of multiple chronic conditions on self-rated health, disability and quality of life among the older populations of Northern Ireland and the Republic of Ireland: a comparison of two nationally representative cross-sectional surveys	Wrong patient population

No.	Authors	Year	Title	Reason for exclusion
163	McIntyre, R. S.; Konarski, J. Z.; Soczynska, J. K.; Wilkins, K.; Panjwani, G.; Bouffard, B.; Bottas, A.; Kennedy, S. H.	2006	Medical comorbidity in bipolar disorder: implications for functional outcomes and health service utilization	Wrong patient population
164	McKinlay, E.; Graham, S.; Horrill, P.	2015	Culturally and linguistically diverse patients' views of multimorbidity and general practice care	Wrong outcomes
165	McKinlay, E.; McDonald, J.; Darlow, B.; Perry, M.	2017	Social networks of patients with multimorbidity: a qualitative study of patients' and supporters' views	Wrong patient population
166	McKinlay, E.; McDonald, J.; Darlow, B.; Perry, M.	2019	The social networks of New Zealand patients with multimorbidity and the work of those nominated as their 'significant supporters': An exploratory study	Wrong patient population
167	Melzer, D.; Gardener, E.; Guralnik, J. M.	2005	Mobility disability in the middle-aged: cross-sectional associations in the English Longitudinal Study of Ageing	Wrong outcomes
168	Mengoni, S. E.; Gates, B.; Parkes, G.; Wellsted, D.; Barton, G.; Ring, H.; Khoo, M. E.; Monji-Patel, D.; Friedli, K.; Zia, A.; Durand, M. A.	2016	"Sometimes, it just stops me from doing anything": A qualitative exploration of epilepsy management in people with intellectual disabilities and their carers	Wrong patient population
169	Merikangas, K. R.; Ames, M.; Cui, L.; Stang, P. E.; Ustun, T. B.; Von Korff, M.; Kessler, R. C.	2007	The impact of comorbidity of mental and physical conditions on role disability in the US adult household population	Wrong patient population
170	Miranda, A.; Berenguer, C.; Colomer, C.; Rosello, R.	2014	Influence of the symptoms of Attention Deficit Hyperactivity Disorder (ADHD) and comorbid disorders on functioning in adulthood	Wrong patient population
171	Moen, V. P.; Drageset, J.; Eide, G. E.; Gjesdal, S.	2018	Dimensions and predictors of disability-A baseline study of patients entering somatic rehabilitation in secondary care	Wrong patient population
172	Mollica, Richard F.; McInnes, Keith; Sarajlic, Narcisa; Lavelle, James; Sarajlic, Iris; Massagli, Michael P.; Mollica, R. F.; McInnes, K.; Sarajlić, N.; Lavelle, J.; Sarajlić, I.; Massagli, M. P.	1999	Disability associated with psychiatric comorbidity and health status in Bosnian refugees living in Croatia	Wrong patient population
173	Mondor, Luke; Maxwell, Colleen; Bronskill, Susan; Gruneir, Andrea; Wodchis, Walter; Maxwell, Colleen J.; Bronskill, Susan E.; Wodchis, Walter P.	2016	The relative impact of chronic conditions and multimorbidity on health-related quality of life in Ontario long-stay home care clients	Wrong patient population

No.	Authors	Year	Title	Reason for exclusion
174	Morales-Asencio, J. M.; Martin-Santos, F. J.; Kaknani, S.; Morilla-Herrera, J. C.; Cuevas Fernandez-Gallego, M.; Garcia-Mayor, S.; Leon-Campos, A.; Morales-Gil, I. M.	2016	Living with chronicity and complexity: Lessons for redesigning case management from patients' life stories - A qualitative study	Wrong patient population
175	Munir, F.; Yarker, J.; McDermott, H.	2009	Employment and the common cancers: correlates of work ability during or following cancer treatment	Wrong study design
176	Nair, Kavita; Ghushchyan, Vahram; Van Den Bos, Jill; Halford, Michael L.; Tan, Gideon; Frech-Tamas, Feride H.; Doyle, Joseph	2012	Burden of Illness for an Employed Population with Chronic Obstructive Pulmonary Disease	Wrong outcomes
177	Nakata, K.; Tsuji, T.; Vietri, J.; Jaffe, D. H.	2018	Work impairment, osteoarthritis, and health-related quality of life among employees in Japan	Wrong patient population
178	Narayanan, S.; Wilson, K.; Ogelsby, A.; Juneau, P.; Durden, E.	2013	Economic burden of systemic lupus erythematosus flares and comorbidities in a commercially insured population in the United States	Wrong patient population
179	Nazari, Goris; Osifeso, Temitope A.; MacDermid, Joy C.	2020	Distribution of Number, Location of Pain and Comorbidities, and Determinants of Work Limitations among Firefighters	Wrong patient population
180	Ngangue, P. A.; Forgues, C.; Nguyen, T.; Sasseville, M.; Gallagher, F.; Loignon, C.; Stewart, M.; Belle Brown, J.; Chouinard, M. C.; Fortin, M.	2020	Patients, caregivers and health-care professionals' experience with an interdisciplinary intervention for people with multimorbidity in primary care: A qualitative study	Wrong outcomes
181	Nguyen, Ruby H. N.; Ecklund, Ali M.; MacLehose, Richard F.; Veasley, Christin; Harlow, Bernard L.	2012	Co-morbid pain conditions and feelings of invalidation and isolation among women with vulvodynia	Wrong patient population
182	Nicholson, K.; Rodrigues, R.; Anderson, K. K.; Wilk, P.; Guaiana, G.; Stranges, S.	2020	Sleep behaviours and multimorbidity occurrence in middle-aged and older adults: findings from the Canadian Longitudinal Study on Aging (CLSA)	Wrong outcomes
183	Nickerson, Angela; Schick, Matthis; Schnyder, Ulrich; Bryant, Richard A.; Morina, Naser	2017	Comorbidity of posttraumatic stress disorder and depression in tortured, treatment-seeking refugees	Wrong outcomes
184	Nikiphorou, Elena; Guh, Daphne; Bansback, Nick; Zhang, Wei; Dixey, Josh; Williams, Peter; Young, Adam	2012	Work disability rates in RA. Results from an inception cohort with 24 years follow-up	Wrong patient population

No.	Authors	Year	Title	Reason for exclusion
185	Norberg, Melissa M.; Diefenbach, Gretchen J.; Tolin, David F.	2008	Quality of life and anxiety and depressive disorder comorbidity	Wrong outcomes
186	Okabe, T.; Abe, Y.; Tomita, Y.; Mizukami, S.; Kanagae, M.; Arima, K.; Nishimura, T.; Tsujimoto, R.; Tanaka, N.; Goto, H.; Horiguchi, I.; Aoyagi, K.	2017	Age-specific risk factors for incident disability in activities of daily living among middle-aged and elderly community-dwelling Japanese women during an 8-9-year follow up: The Hizen-Oshima study	Wrong patient population
187	Oldridge, N. B.; Stump, T. E.	2004	Heart disease, comorbidity, and activity limitation in community-dwelling elderly	Wrong patient population
188	O'Neil, A.; Williams, E. D.; Stevenson, C. E.; Oldenburg, B.; Sanderson, K.	2012	Co-morbid depression is associated with poor work outcomes in persons with cardiovascular disease (CVD): a large, nationally representative survey in the Australian population	Wrong patient population
189	Ouimette, P. C.; Gima, K.; Moos, R. H.; Finney, J. W.	1999	A comparative evaluation of substance abuse treatment IV. The effect of comorbid psychiatric diagnoses on amount of treatment, continuing care, and 1-year outcomes	Wrong patient population
190	Øverland, S.; Harvey, S. B.; Knudsen, A. K.; Mykletun, A.; Hotopf, M.	2012	Widespread pain and medically certified disability pension in the Hordaland Health Study	Wrong patient population
191	Owsley, C.; McGwin, G.	2007	Measuring the personal burden of eye disease and vision impairment	Wrong patient population
192	Paddison, Charlotte A. M.; Saunders, Catherine L.; Abel, Gary A.; Payne, Rupert A.; Adler, Amanda I.; Graffy, Jonathan P.; Roland, Martin O.	2015	How do people with diabetes describe their experiences in primary care? Evidence from 85,760 patients with self-reported diabetes from the English General Practice Patient Survey	Wrong outcomes
193	Parker, Gordon; Rosen, Alan; Trauer, Tom; Hadzi-Pavlovic, Dusan	2007	Disability associated with mood states and comparator conditions: Application of the Life Skills Profile measure of disability	Wrong patient population
194	Parker, K. M.; Wilson, M. G.; Vandenberg, R. J.; DeJoy, D. M.; Orpinas, P.	2009	Association of Comorbid Mental Health Symptoms and Physical Health Conditions With Employee Productivity	Wrong patient population
195	Parker, W. M.; Ferreira, K.; Vernon, L.; Cardone, K. E.	2017	The delicate balance of keeping it all together: Using social capital to manage multiple medications for patients on dialysis	Wrong patient population

No.	Authors	Year	Title	Reason for exclusion
196	Parrish, M. M.; Adams, S.	2003	Caregiver comorbidity and the ability to manage stress	Wrong patient population
197	Patterson, K. K.; Sibley, K. M.	2016	Arthritis and associated limitations in community-dwelling Canadians living with stroke	Wrong patient population
198	Pelletier, L.; Shanmugasegaram, S.; Patten, S. B.; Demers, A.	2017	Self-management of mood and/or anxiety disorders through physical activity/exercise	Wrong outcomes
199	Person, B.; Addiss, D.; Bartholomew, L. K.; Meijer, C.; Pou, V.; Gonzálvez, G.; Borne, B. V.	2008	'Can it be that God does not remember me': a qualitative study on the psychological distress, suffering, and coping of Dominican women with chronic filarial lymphedema and elephantiasis of the leg	Wrong patient population
200	Peters-Klimm, F.; Freund, T.; Kunz, C. U.; Laux, G.; Frankenstein, L.; Muller-Tasch, T.; Szecsenyi, J.	2013	Determinants of heart failure self-care behaviour in community-based patients: a cross-sectional study	Wrong patient population
201	Plantinga, L. C.; Johansen, K.; Crews, D. C.; Shahinian, V. B.; Robinson, B. M.; Saran, R.; Burrows, N. R.; Williams, D. E.; Powe, N. R.; Cdc Ckd Surveillance Team	2011	Association of CKD with disability in the United States	Wrong patient population
202	Polenick, C. A.; Leggett, A. N.; Webster, N. J.; Han, B. H.; Zarit, S. H.; Piette, J. D.	2020	Multiple Chronic Conditions in Spousal Caregivers of Older Adults With Functional Disability: Associations With Caregiving Difficulties and Gains	Wrong patient population
203	Prazeres, F.; Santiago, L.	2016	Relationship between health-related quality of life, perceived family support and unmet health needs in adult patients with multimorbidity attending primary care in Portugal: a multicentre cross-sectional study	Wrong outcomes
204	Qin, J.; Theis, K. A.; Barbour, K. E.; Helmick, C. G.; Baker, N. A.; Brady, T. J.; Centers for Disease, Control; Prevention,	2015	Impact of arthritis and multiple chronic conditions on selected life domains - United States, 2013	Wrong publication type (e.g. conference proceeding)
205	Rankin, Sally H.	1995	Going it alone: Women managing recovery from acute myocardial infarction	Wrong patient population

No.	Authors	Year	Title	Reason for exclusion
206	Razzano, Lisa A.; Hamilton, Marie M.; Yost, Chantelle; Pashka, Nicole J.; Perloff, Judith K.	2015	Employment outcomes for individuals with HIV/AIDS and co-occurring mental health factors	Wrong outcomes
207	Restorick Roberts, A.; Betts Adams, K.; Beckette Warner, C.	2017	Effects of chronic illness on daily life and barriers to self-care for older women: A mixed-methods exploration	Wrong patient population
208	Reynolds, R.; Hurley, S.; Torres, M.; Jackson, J.; Boyd, P.; Chen, V. W.	2000	Use of coping strategies and breast cancer survival: results from the Black/White Cancer Survival Study	Wrong patient population
209	Riegel, B.; Carlson, B.	2002	Facilitators and barriers to heart failure self-care	Wrong patient population
210	Rijken, Mieke; Valderas, José Maria; Heins, Marianne; Schellevis, Francois; Korevaar, Joke	2020	Identifying high-need patients with multimorbidity from their illness perceptions and personal resources to manage their health and care: a longitudinal study	Wrong patient population
211	Rijken, Mieke; van der Heide, Iris	2019	Identifying subgroups of persons with multimorbidity based on their needs for care and support	Wrong patient population
212	Rivera, J.; Esteve-Vives, J.; Vallejo, M. A.; Rejas, J.; Grupo, Icaf	2011	Factors associated with temporary work disability in patients with fibromyalgia	Wrong patient population
213	Rizvi, Sakina J.; Cyriac, Anna; Grima, Etienne; Tan, Mary; Lin, Peter; Gallaugher, Laura Ashley; McIntyre, Roger S.; Kennedy, Sidney H.	2015	Depression and employment status in primary and tertiary care settings	Wrong patient population
214	Rodriguez-Salgado, B.; Dolengevich-Segal, H.; Arrojo-Romero, M.; Castelli-Candia, P.; Navio-Acosta, M.; Perez-Rodriguez, M. M.; Saiz-Ruiz, J.; Baca-Garcia, E.	2006	Perceived quality of life in obsessive-compulsive disorder: related factors	Wrong outcomes
215	Rohrbacker, N. J.; Kleinman, N. L.; White, S. A.; March, J. L.; Reynolds, M. R.	2010	The burden of atrial fibrillation and other cardiac arrhythmias in an employed population: associated costs, absences, and objective productivity loss	Wrong patient population
216	Ropponen, A.; Gemes, K.; Frumento, P.; Almondo, G.; Bottai, M.; Friberg, E.; Alexanderson, K.	2020	Predicting the duration of sickness absence spells due to back pain: a population-based study from Sweden	Wrong outcomes
217	Rosbach, M.; Andersen, J. S.	2017	Patient-experienced burden of treatment in patients with multimorbidity - A systematic review of qualitative data	Wrong study design

No.	Authors	Year	Title	Reason for exclusion
218	Sabaz, M.; Simpson, G. K.; Walker, A. J.; Rogers, J. M.; Gillis, I.; Strettles, B.	2014	Prevalence, comorbidities, and correlates of challenging behavior among community-dwelling adults with severe traumatic brain injury: a multicenter study	Wrong patient population
219	Sangalli, V.; Dukes, J.; Doppalapudi, S. B.; Costa, G.; Neri, L.	2014	Work Ability and Labor Supply after Kidney Transplantation	Wrong patient population
220	Sareen, Jitender; Jacobi, Frank; Cox, Brian J.; Belik, Shay-Lee; Clara, Ian; Stein, Murray B.	2006	Disability and poor quality of life associated with comorbid anxiety disorders and physical conditions (PSYNDEXshort)	Wrong patient population
221	Saunders, K.; Merikangas, K.; Low, N. C.; Von Korff, M.; Kessler, R. C.	2008	Impact of comorbidity on headache-related disability	Wrong outcomes
222	Sav, Adem; King, Michelle A.; Whitty, Jennifer A.; Kendall, Elizabeth; McMillan, Sara S.; Kelly, Fiona; Hunter, Beth; Wheeler, Amanda J.	2015	Burden of treatment for chronic illness: a concept analysis and review of the literature	Wrong study design
223	Schmitz, N.; Wang, J.; Lesage, A.; Malla, A.; Strychar, I.	2008	Psychological distress and short-term disability in people with diabetes: results from the Canadian Community Health Survey	Wrong patient population
224	Schofield, Deborah J.; Callander, Emily J.; Shrestha, Rupendra N.; Passey, Megan E.; Percival, Richard; Kelly, Simon J.	2012	Association between co-morbidities and labour force participation amongst persons with back problems	Wrong patient population
225	Schoppen, T.; Boonstra, A.; Groothoff, J. W.; De Vries, J.; Goeken, L. N.; Eisma, W. H.	2002	Job satisfaction and health experience of people with a lower-limb amputation in comparison with healthy colleagues	Wrong patient population
226	Schultz, A. B.; Edington, D. W.	2009	Metabolic syndrome in a workplace: prevalence, co-morbidities, and economic impact	Wrong patient population
227	Scott, K. M.; Von Korff, M.; Alonso, J.; Angermeyer, M. C.; Bromet, E.; Fayyad, J.; de Girolamo, G.; Demyttenaere, K.; Gasquet, I.; Gureje, O.; Haro, J. M.; He, Y.; Kessler, R. C.; Levinson, D.; Medina Mora, M. E.; Oakley Browne, M.; Ormel, J.; Posada-Villa, J.; Watanabe, M.; Williams, D.	2009	Mental-physical co-morbidity and its relationship with disability: results from the World Mental Health Surveys	Wrong patient population
228	Serrano, V.; Spencer-Bonilla, G.; Boehmer, K. R.; Montori, V. M.	2017	Minimally Disruptive Medicine for Patients with Diabetes	Wrong study design

No.	Authors	Year	Title	Reason for exclusion
229	Shadloo, Behrang; Amin-Esmaeili, Masoumeh; Motevalian, Abbas; Mohraz, Minoo; Sedaghat, Abbas; Gouya, Mohammad Mehdi; Rahimi-Movaghar, Afarin	2018	Psychiatric disorders among people living with HIV/AIDS in Iran: Prevalence, severity, service utilization and unmet mental health needs	Wrong outcomes
230	Shelby, R. A.; Golden-Kreutz, D. M.; Andersen, B. L.	2008	PTSD diagnoses, subsyndromal symptoms, and comorbidities contribute to impairments for breast cancer survivors	Wrong patient population
231	Shiri, Rahman; Kaila-Kangas, Leena; Ahola, Kiris; Kivekäs, Teija; Viikari-Juntura, Eira; Heliövaara, Markuu; Miranda, Helena; Leino-Arjas, Pöivi	2013	The Relation of Co-occurring Musculoskeletal Pain and Depressive Symptoms With Work Ability	Wrong patient population
232	Signal, L.; Semper, K.; Stairmand, J.; Davies, C.; Millar, E.; Dowell, T.; Lawrenson, R.; Mangin, D.; Sarfati, D.	2017	A walking stick in one hand and a chainsaw in the other: patients' perspectives of living with multimorbidity	Wrong patient population
233	Sim, Kang; Chan, Yiong Huak; Chua, Thiam Hee; Mahendran, Rathi; Chong, Siow Ann; McGorry, Patrick	2006	Physical comorbidity, insight, quality of life and global functioning in first episode schizophrenia: A 24-month, longitudinal outcome study	Wrong patient population
234	Simmonds, R. L.; Tylee, A.; Walters, P.; Rose, D.	2013	Patients' perceptions of depression and coronary heart disease: a qualitative UPBEAT-UK study	Wrong patient population
235	Simon, G. E.; Revicki, D.; Heiligenstein, J.; Grothaus, L.; VonKorff, M.; Katon, W. J.; Hylan, T. R.	2000	Recovery from depression, work productivity, and health care costs among primary care patients	Wrong patient population
236	Slightam, Cindie A.; Brandt, Kirsten; Jenchura, Emily C.; Lewis, Eleanor T.; Asch, Steven M.; Zulman, Donna M.	2018	'I had to change so much in my life to live with my new limitations': Multimorbid patients' descriptions of their most bothersome chronic conditions	Wrong patient population
237	Stanley, J.; Millar, E.; Semper, K.; Davies, C.; Dowell, A.; Mangin, D.; Lawrenson, R.; Sarfati, D.	2018	The impact of multimorbidity on people's lives: a cross-sectional survey	Wrong patient population
238	Stein, M. B.; Cox, B. J.; Afifi, T. O.; Belik, S. L.; Sareen, J.	2006	Does co-morbid depressive illness magnify the impact of chronic physical illness? A population-based perspective	Wrong patient population
239	Stein, M. B.; Heimberg, R. G.	2004	Well-being and life satisfaction in generalized anxiety disorder: comparison to major depressive disorder in a community sample	Wrong patient population

No.	Authors	Year	Title	Reason for exclusion
240	Stein, M. B.; Kean, Y. M.	2000	Disability and quality of life in social phobia: epidemiologic findings	Wrong patient population
241	Stuifbergen, A.; Brown, A.; Phillips, L.	2009	Predictors and moderators of the disablement process in persons with multiple sclerosis	Wrong patient population
242	Stull, D. E.; Starling, R.; Haas, G.; Young, J. B.	1999	Becoming a patient with heart failure	Wrong patient population
243	Styron, J. F.; Barsoum, W. K.; Smyth, K. A.; Singer, M. E.	2011	Preoperative predictors of returning to work following primary total knee arthroplasty	Wrong patient population
244	Sum, G.; Ishida, M.; Koh, G. C.; Singh, A.; Oldenburg, B.; Lee, J. T.	2020	Implications of multimorbidity on healthcare utilisation and work productivity by socioeconomic groups: Cross-sectional analyses of Australia and Japan	Wrong patient population
245	Sumelahti, M. L.; Sumanen, M.; Sumanen, M. S.; Tuominen, S.; Vikkula, J.; Honkala, S. M.; Rosqvist, S.; Korolainen, M. A.	2020	My Migraine Voice survey: disease impact on healthcare resource utilization, personal and working life in Finland	Wrong patient population
246	Sunderland, M.; Newby, J. M.; Andrews, G.	2013	Health anxiety in Australia: prevalence, comorbidity, disability and service use	Wrong patient population
247	Sundstrup, E.; Jakobsen, M. D.; Mortensen, O. S.; Andersen, L. L.	2017	Joint association of multimorbidity and work ability with risk of long-term sickness absence: a prospective cohort study with register follow-up	Wrong patient population
248	Taylor, W. J.	2012	Impact of psoriatic arthritis on the patient: through the lens of the WHO International Classification of Functioning, Health, and Disability	Wrong study design
249	Terauchi, Y.; Ozaki, A.; Zhao, X.; Teoh, C.; Jaffe, D.; Tajima, Y.; Shuto, Y.	2019	Humanistic and economic burden of cardiovascular disease related comorbidities and hypoglycaemia among patients with type 2 diabetes in Japan	Wrong patient population
250	Tollefson, G. D.; Souetre, E.; Thomander, L.; Potvin, J. H.	1993	Comorbid anxious signs and symptoms in major depression: impact on functional work capacity and comparative treatment outcomes	Wrong outcomes
251	Townsend, A.; Hunt, K.; Wyke, S.	2003	Managing multiple morbidity in mid-life: a qualitative study of attitudes to drug use	Wrong outcomes

No.	Authors	Year	Title	Reason for exclusion
252	Umeh, Nkeiruka I.; Ajegba, Brittany; Buscetta, Ashley J.; Abdallah, Khadijah E.; Minniti, Caterina P.; Bonham, Vence L.	2017	The psychosocial impact of leg ulcers in patients with sickle cell disease: I don't want them to know my little secret	Wrong patient population
253	Vamos, E. P.; Mucsi, I.; Keszei, A.; Kopp, M. S.; Novak, M.	2009	Comorbid Depression Is Associated With Increased Healthcare Utilization and Lost Productivity in Persons With Diabetes: A Large Nationally Representative Hungarian Population Survey	Wrong patient population
254	van den Berg, S.; Burdorf, A.; Robroek, S. J. W.	2017	Associations between common diseases and work ability and sick leave among health care workers	Wrong patient population
255	van der Burg, L. R.; Boonen, A.; van Amelsvoort, L. G.; Jansen, N. W.; Landewe, R. B.; Kant, I.	2014	Effects of cardiovascular comorbidities on work participation in rheumatic diseases: a prospective cohort study among working individuals	Wrong patient population
256	van der Werff, E.; Verboom, C. E.; Penninx, B. W.; Nolen, W. A.; Ormel, J.	2010	Explaining heterogeneity in disability associated with current major depressive disorder: effects of illness characteristics and comorbid mental disorders	Wrong patient population
257	Van Duin, M. J.; Conde, R.; Wijnen, B.; Evers, S. M.; Gonzalez-Rodriguez, J. L.; Govers, M. J.; Hiligsmann, M.	2017	The impact of comorbidities on costs, utilities and health-related quality of life among HIV patients in a clinical setting in Bogota	Wrong patient population
258	van Merode, T.; van de Ven, K.; van den Akker, M.	2018	Patients with multimorbidity and their treatment burden in different daily life domains: a qualitative study in primary care in the Netherlands and Belgium	Wrong patient population
259	van Zon, Sander K. R.; Reijneveld, Sijmen A.; Galaurchi, Anne; de Leon, Carlos F. Mendes; Almansa, Josué; Bültmann, Ute	2020	Multimorbidity and the transition out of full-time paid employment: A longitudinal analysis of the Health and Retirement Study	Wrong patient population
260	Verbrugge, L. M.; Lepkowski, J. M.; Konkol, L. L.	1991	Levels of disability among U.S. adults with arthritis	Wrong patient population
261	Veronese, A.; Ayuso-Mateos, J. L.; Cabello, M.; Chatterji, S.; Nuevo, R.	2012	Work disability and depressive disorders: impact on the European population	Wrong patient population
262	Vietri, J.; Otsubo, T.; Montgomery, W.; Tsuji, T.; Harada, E.	2015	The incremental burden of pain in patients with depression: results of a Japanese survey	Wrong patient population
263	Volker, Deborah L; Becker, Heather; Kang, Sook Jung; Kullberg, Vicki	2013	A double whammy: health promotion among cancer survivors with preexisting functional limitations	Wrong patient population

No.	Authors	Year	Title	Reason for exclusion
264	Vollman, M. W.; Lamontagne, L. L.; Hepworth, J. T.	2007	Coping and depressive symptoms in adults living with heart failure	Wrong outcomes
265	Von Korff, M.; Katon, W. J.; Lin, E. H.; Ciechanowski, P.; Peterson, D.; Ludman, E. J.; Young, B.; Rutter, C. M.	2011	Functional outcomes of multi-condition collaborative care and successful ageing: results of randomised trial	Wrong patient population
266	Waghorn, G.; Chant, D.	2006	Work performance among Australians with depression and anxiety disorders: a population level second order analysis	Wrong patient population
267	Waghorn, G.; Chant, D.; Jonsdottir, A.	2011	Comorbidity and Labor Force Activity Among People With Psychiatric Disorders	Wrong patient population
268	Waghorn, G.; Lloyd, C.; Abraham, B.; Silvester, D.; Chant, D.	2008	Comorbid physical health conditions hinder employment among people with psychiatric disabilities	Wrong patient population
269	Waghorn, Geoff; Chant, David; Lloyd, Chris	2006	Labor force activity among Australians with musculoskeletal disorders comorbid with depression and anxiety disorders	Wrong patient population
270	Waghorn, Geoffrey; Chant, David	2011	Receiving Treatment, Labor Force Activity, and Work Performance Among People with Psychiatric Disorders: Results from a Population Survey	Wrong patient population
271	Wang, R.; Zhao, Y.; He, X.; Ma, X.; Yan, X.; Sun, Y.; Liu, W.; Gu, Z.; Zhao, J.; He, J.	2009	Impact of hypertension on health-related quality of life in a population-based study in Shanghai, China	Wrong outcomes
272	Ward, B. W.	2015	Multiple chronic conditions and labor force outcomes: A population study of U.S. adults	Wrong patient population
273	Warner, Lisa M.; Schüz, Benjamin; Wurm, Susanne; Ziegelmann, Jochen P.; Tesch-Römer, Clemens	2010	Giving and taking-Differential effects of providing, receiving and anticipating emotional support on quality of life in adults with multiple illnesses	Wrong patient population
274	Wedegaertner, Felix; Arnhold-Kerri, Sonja; Sittaro, Nicola-Alexander; Bleich, Stefan; Geyer, Siegfried; Lee, William E.	2013	Depression- and anxiety-related sick leave and the risk of permanent disability and mortality in the working population in Germany: a cohort study	Wrong patient population

No.	Authors	Year	Title	Reason for exclusion
275	Weldam, S. W.; Lammers, J. W.; Decates, R. L.; Schuurmans, M. J.	2013	Daily activities and health-related quality of life in patients with chronic obstructive pulmonary disease: psychological determinants: a cross-sectional study	Wrong patient population
276	Westhoff, G.; Listing, J.; Zink, A.	2000	Loss of physical independence in rheumatoid arthritis: interview data from a representative sample of patients in rheumatologic care	Wrong patient population
277	Whiteley, J.; Emir, B.; Seitzman, R.; Makinson, G.	2016	The burden of atopic dermatitis in US adults: results from the 2013 National Health and Wellness Survey	Wrong patient population
278	Whitson, H. E.; Steinhauser, K.; Ammarell, N.; Whitaker, D.; Cousins, S. W.; Ansah, D.; Sanders, L. L.; Cohen, H. J.	2011	Categorizing the effect of comorbidity: a qualitative study of individuals' experiences in a low-vision rehabilitation program	Wrong patient population
279	Wilkie, R.; Peat, G.; Thomas, E.; Croft, P.	2007	Factors associated with restricted mobility outside the home in community-dwelling adults ages fifty years and older with knee pain: an example of use of the International Classification of Functioning to investigate participation restriction	Wrong patient population
280	Wilkie, R.; Phillipson, C.; Hay, E.; Pransky, G.	2014	Frequency and predictors of premature work loss in primary care consulters for osteoarthritis: prospective cohort study	Wrong patient population
281	Wilson-Genderson, M.; Heid, A. R.; Pruchno, R.	2017	Onset of Multiple Chronic Conditions and Depressive Symptoms: A Life Events Perspective	Wrong outcomes
282	Wittchen, H. U.; Fuetsch, M.; Sonntag, H.; Muller, N.; Liebowitz, M.	1999	Disability and quality of life in pure and comorbid social phobia--findings from a controlled study	Duplicate
283	Wu, C. H.; Erickson, S. R.	2012	The association between asthma and absenteeism among working adults in the United States: results from the 2008 medical expenditure panel survey	Wrong patient population
284	Yang, R.; Wang, J.; Wang, H.; Tracy, E. L.; Tracy, C. T.	2020	A cross-lagged model of depressive symptoms and mobility disability among middle-aged and older Chinese adults with arthritis	Wrong patient population
285	Zhang, Amy Y; Gary, Faye; Zhu, Hui	2015	Exploration of depressive symptoms in African American cancer patients	Wrong outcomes
286	Zhang, Wei; Koehoorn, Mieke; Anis, Aslam H.	2010	Work productivity among employed Canadians with arthritis	Wrong patient population

No.	Authors	Year	Title	Reason for exclusion
287	Zhou, C. H.; Tang, S. F.; Wang, X. H.; Chen, Z.; Zhang, D. I.; Gao, J. L.; Ghose, B.; Feng, D.; He, Z. F.; Yaya, S.; Feng, Z. C.	2018	Satisfaction about Patient-centeredness and Healthcare System among Patients with Chronic Multimorbidity	Wrong outcomes
288	Zulman, D. M.; Jenchura, E. C.; Cohen, D. M.; Lewis, E. T.; Houston, T. K.; Asch, S. M.	2015	How Can eHealth Technology Address Challenges Related to Multimorbidity? Perspectives from Patients with Multiple Chronic Conditions	Wrong outcomes
