# Supplementary material for: Everyday Lives of Middle-Aged Persons with Multimorbidity: A Mixed Methods Systematic Review
Source: Int J Environ Res Public Health. 2021 Dec 21;19(1):6. doi: 10.3390/ijerph19010006 (PMC8751163; doi:10.3390/ijerph19010006)
Supplement: Supplementary file 1 [file ijerph-19-00006-s001.zip › Supplementary files/Table S2.pdf]

Table S2. Search for everyday life experiences of middle-aged persons with multimorbidity

10.12.2020 – Medline [medall] via Ovid

1	(Comorbid* or co-morbid* or multimorbid* or multi-morbid* or multiple morbid* or multiple diseases or multiple chronic conditions or multiple chronic diseases or multiple chronic morbidities or multiple long-term conditions or multiple long-term health conditions).ti,ab,kf.	199,674
2	exp Comorbidity/	112,002
3	Multiple Chronic Conditions/	491
4	Middle Aged/	4,417,485
5	Adult/	5,064,713
6	(2 or 3) and (4 or 5)	69,046
7	1 or 6	240,361
8	(normal life or everyday life or daily life).ti,ab,kf.	33,184
9	(family or friends or relatives).ti,ab,kf.	883619
10	(work life or employment or unemploy* or sick leave days or productivity or work capacity).ti,ab,kf.	140,890
11	(leisure or spare time or free time or hobby or hobbies or private life or social life).ti,ab,kf.	24,911
12	Work Performance/	908
13	Social Environment/	43,077
14	exp "Activities of Daily Living"/	104,013
15	exp Leisure Activities/	240,547
16	or/8-15	1,407,335
17	(disparit* or barrier* or challenge* or discrimination or disadvantage* or disabilit* or handicap* or financial difficult* or financial problem*).ti,ab,kf.	1,422,546
18	(coping strateg* or abilities or capacities or coaching or resilience or motivation or facilitator* or financial protection or material resources or telemonitoring application or home-based or web-based or mobile health tool*).ti,ab,kf.	310,612
19	(experience* or perceive* or perspective* or preference*).ti,ab,kf.	1,702,742
20	Financial Support/	3,806
21	Internet-Based Intervention/	309
22	Adaptation, Psychological/	95,566
23	Social Support/	71,764
24	or/17-23	3,210,947
25	(health care or healthcare or health system or health service* or community or public policy or organization* or government* or business or rehabilitative care or care provider* or care management or support or social network or workplace or employer* or company or district or neighborhood or neighbourhood).ti,ab,kf.	2,513,804
26	Occupational Health Services/	10,585
27	Occupational Health/	34,007
28	exp Community Health Services/	307,375
29	Health Services for Persons with Disabilities/	128
30	Social Work, Psychiatric/	2,684
31	Personal Health Services/	1,946
32	Preventive Health Services/	13,657

33	Social Work/	15,404
34	Urban Health Services/	3,679
35	exp Patient-Centered Care/	20,825
36	Self-Help Groups/	9,173
37	Health Planning Organizations/	893
38	or/25-37	2,740,112
39	(living with multimorbidity or living with multiple chronic conditions or living with multiple chronic diseases or coping with multimorbidity or coping with multiple chronic conditions or coping with multiple chronic diseases).ti,ab,kf.	89
40	(7 and 16 and 24 and 38) or 39	3,869
