# Supplementary material for: Everyday Lives of Middle-Aged Persons with Multimorbidity: A Mixed Methods Systematic Review
Source: Int J Environ Res Public Health. 2021 Dec 21;19(1):6. doi: 10.3390/ijerph19010006 (PMC8751163; doi:10.3390/ijerph19010006)
Supplement: Supplementary file 1 [file ijerph-19-00006-s001.zip › Supplementary files/Table S4.pdf]

Table S4. Risk of bias assessment of included studies using the Mixed Methods Appraisal Tool (MMAT)

Author, Year (Reference)	1. Qualitative studies				
	1.1. Is the qualitative approach appropriate to answer the research question?	1.2. Are the qualitative data collection methods suitable for addressing the research question?	1.3. Are the findings adequately derived from the data?	1.4. Is the interpretation of results sufficiently substantiated by data?	1.5. Are qualitative data sources, collection, analysis and interpretation coherent?
Cheng, 2019 (1)	Yes	Yes	Yes	Yes	Yes
Morgan, 2019 (2)	Yes	Yes	Yes	Yes	Yes
Noël, 2005 (3)	Yes	Yes	Yes	Yes	Yes
O'Brien, 2014 (4)	Yes	Yes	Yes	Yes	Yes
Ørtenblad, 2018 (5)	Yes	Yes	Yes	Yes	Yes
Ridgeway, 2014 (6)	Yes	Yes	Yes	Yes	Yes
Sand, 2021 (7)	Yes	Yes	Yes	Yes	Yes
Slomka, 2017 (8)	Yes	Yes	Yes	Yes	Yes
Subramanian, 2017 (9)	Yes	Yes	Yes	Yes	Yes
Townsend, 2003 (10)	Yes	Yes	Yes	Yes	Yes
Warren-Jeanpiere, 2014 (11)	Yes	Yes	Yes	Yes	Yes
White, 2016# (12)	Yes	Yes	Yes	Yes	Yes
White, 2019# (12)	Yes	Yes	Yes	Yes	Yes
**Author, Year (Reference)**	**3. Quantitative descriptive studies**				
	4.1. Is the sampling strategy relevant to	4.2. Is the sample representative of the target population?	4.3. Are the measurements appropriate?	4.4. Is the risk of non-response bias low?	4.5. Is the statistical analysis appropriate to

	address the research question?				answer the research question?
Arnold, 2016 (13)	Yes	No	Yes	Can't tell	Yes
Bell, 2004 (14)	Yes	Can't tell	Yes	No	Yes
Buckner, 2008 (15)	Yes	Yes	Yes	Yes	Yes
Conover, 2006 (16)	Yes	No	Yes	Can't tell	Yes
Dagher, 2015 (17)	Yes	Yes	Yes	Can't tell	Yes
D'Amico, 2018 (18)	Yes	Yes	Yes	Can't tell	Yes
Deschenes, 2015 (19)	Yes	No	Yes	Can't tell	Yes
Díaz, 2016 (20)	Yes	No	Yes	Can't tell	Yes
Dutton, 2014 (21)	Yes	No	Yes	Can't tell	Yes
Egede, 2007 (22)	Yes	No	Yes	Can't tell	Yes
Gehrke, 2017 (23)	Yes	No	Yes	Can't tell	Yes
Gulley, 2011 (24)	Yes	Can't tell	Yes	Can't tell	Yes
Hakola, 2011 (25)	Yes	Yes	Yes	Can't tell	Yes
Johs, 2017 (26)	Yes	Can't tell	Yes	Can't tell	Yes
Li, 2019 (27)	Yes	Yes	Yes	Yes	Yes
Linder, 2009 (28)	Yes	Yes	Yes	Can't tell	Yes
Motl, 2011 (29)	Yes	No	Yes	Can't tell	Yes
Neri, 2009 (30)	Yes	Yes	Yes	Yes	Yes
Nikiphorou, 2017 (31)	Yes	Can't tell	Yes	Can't tell	Yes
Rao, 2015 (32)	Yes	Yes	Yes	Can't tell	Yes
Romera, 2011 (33)	Yes	No	Yes	Can't tell	Yes
Saris, 2017 (34)	Yes	Can't tell	Yes	Can't tell	Yes
Schofield, 2014 (35)	Yes	Can't tell	Yes	Can't tell	Yes
Schonauer, 1999 (36)	Yes	Yes	Yes	Can't tell	Yes
Souêtre, 1994 (37)	Yes	Yes	Yes	Can't tell	Yes

Tian, 2005 (38)	Yes	No	Yes	Can't tell	Yes
Todd, 2004 (39)	Yes	No	Yes	Can't tell	Yes
Weijman, 2004 (40)	Yes	No	Yes	Can't tell	Yes
Wittchen, 1999 (41)	Yes	Can't tell	Yes	Can't tell	Yes
Wittchen, 2000 (42)	Yes	Yes	Yes	Yes	Yes
**Author, Year (Reference)**	**4. Mixed methods**				
	5.1. Is the rationale for using a mixed method design to address the research question convincing?	5.2. Are the different components of the study effectively integrated?	5.3. Are the outputs from the integration of qualitative and quantitative components adequately interpreted?	5.4. Are divergences and inconsistencies between quantitative and qualitative results adequately addressed?	5.5. Do the different components of the study adhere to the standard quality criteria for each of the methods involved?
Dickson, 2013 (43)	Yes	Yes	Yes	Yes	Yes

Can't tell = Author did not provide enough information for an assessment.
